# Plastic, It’s What’s for Dinner: A Preliminary Comparison of Ingested Particles in Bottlenose Dolphins and Their Prey

**DOI:** 10.3390/oceans4040028

**Published:** 2023-12-07

**Authors:** Leslie B. Hart, Miranda Dziobak, Randall S. Wells, Elizabeth Berens McCabe, Eric Conger, Tita Curtin, Maggie Knight, John Weinstein

**Affiliations:** 1 Department of Health and Human Performance, School of Health Sciences, College of Charleston, Charleston, SC 29424, USA; 2 Center for Coastal Environmental and Human Health, College of Charleston, Charleston, SC 29424, USA; 3 Department of Environmental Health Sciences, Arnold School of Public Health, University of South Carolina, Columbia, SC 29208, USA; 4 Chicago Zoological Society’s Sarasota Dolphin Research Program, c/o Mote Marine Laboratory, Sarasota, FL 34236, USA; 5 Department of Biology, School of Sciences, Mathematics, and Engineering, College of Charleston, Charleston, SC 29424, USA; 6 Graduate Program in Marine Biology, Grice Marine Laboratory, College of Charleston, Charleston, SC 29424, USA; 7 Department of Biology, The Citadel, Charleston, SC 29409, USA

**Keywords:** plastic pollution, OneHealth, contaminant, plasticizers, seafood safety, marine mammal, cetacean, fish

## Abstract

Microplastic ingestion was reported for common bottlenose dolphins (*Tursiops truncatus*) inhabiting Sarasota Bay, FL, USA, a community that also has prevalent exposure to plasticizers (i.e., phthalates) at concentrations higher than human reference populations. Exposure sources are currently unknown, but plastic-contaminated prey could be a vector. To explore the potential for trophic exposure, prey fish muscle and gastrointestinal tract (GIT) tissues and contents were screened for suspected microplastics, and particle properties (e.g., color, shape, surface texture) were compared with those observed in gastric samples from free-ranging dolphins. Twenty-nine fish across four species (hardhead catfish, *Ariopsis felis*; pigfish, *Orthopristis chrysoptera*; pinfish, *Lagodon rhomboides*; and Gulf toadfish, *Opsanus beta*) were collected from Sarasota Bay during September 2022. Overall, 97% of fish (*n* = 28) had suspected microplastics, and GIT abundance was higher than muscle. Fish and dolphin samples contained fibers and films; however, foams were common in dolphin samples and not observed in fish. Suspected tire wear particles (TWPs) were not in dolphin samples, but 23.1% and 32.0% of fish muscle and GIT samples, respectively, contained at least one suspected TWP. While some similarities in particles were shared between dolphins and fish, small sample sizes and incongruent findings for foams and TWPs suggest further investigation is warranted to understand trophic transfer potential.

## 1. Introduction

Marine plastic pollution is a massive environmental concern. According to a recent study, the world’s oceans contain approximately 171 trillion plastic particles, and considering global dependence on plastic items, inadequate waste management practices, and policy inaction, the amount of marine plastic pollution could nearly triple by 2040 [1,2]. Additionally, the properties that garnered popular interest in plastic (e.g., durability, affordability) contribute to the pervasiveness and ubiquity of this pollutant. Plastic pollution has been identified in every ocean on the planet [3] and across multiple marine taxa [4,5].

Marine plastic debris is often categorized by size [6]. Macro- and mesoplastics (≥5 mm diameter), which may enter the marine environment directly as waste, can lethally and sublethally impact the health of large marine vertebrates, such as sea turtles [7] and marine mammals [8–10], due to entanglement or ingestion. Microplastics (<5 mm in diameter) can enter the environment indirectly via inadequate wastewater filtration or fragmentation of larger plastic items [6,11], and the small particle size makes marine fauna particularly vulnerable to microplastic exposure and toxicity. Plastic particle uptake by fish can occur via direct/active consumption, unintentional ingestion, or branchial intrusion [12,13], and particles have been detected in the gills, gastrointestinal tract (contents and digested tissues), liver, and muscle of exposed fish [5,12,14,15]. Microplastic exposure in fish has been widely reported both geographically and taxonomically [1,5,16], but individual, population, and species-level differences in exposure can be influenced by food availability, feeding behavior, degree and nature of plastic pollution, and foraging sense (i.e., visual vs. chemosensory cues; [13]). Given the widespread prevalence of plastic particle detection in fish and other lower trophic organisms, trophic transfer of microplastics to apex predators seems likely. For example, Romeo et al. (2015) observed microplastics in 30–75% of stomachs from several apex pelagic fish species considered to be specialist feeders, suggesting exposure from active consumption of contaminated prey [17].

Recently, microplastic ingestion was documented in free-ranging common bottlenose dolphins (*Tursiops truncatus*) inhabiting Sarasota Bay, FL, USA [18]. This dolphin community also has prevalent exposure to plasticizers (i.e., phthalates; [19,20]) at levels higher than human reference populations [21]. The source of Sarasota Bay bottlenose dolphin plastic and plasticizer exposure remains unknown, but we hypothesize that plastic-contaminated prey fish could be a possible vector. The objective of this study was to screen for microplastics in muscle and gastrointestinal tracts from bottlenose dolphin prey fish collected in Sarasota Bay and compare particle properties (e.g., color, shape, surface texture) to those observed in gastric samples from bottlenose dolphins. In addition to providing insight on a source of xenobiotic exposure in dolphins, this study may also reveal risks to seafood safety due to trophic concurrence with coastal human communities, given that dolphins and humans are at similar trophic levels [22].

## 2. Materials and Methods

### 2.1. Fish Collection and Sampling

Common prey fish for Sarasota Bay bottlenose dolphins have been identified through examination of the stomach contents of stranded dolphins and field observation [23–25]. Resident dolphins are considered selective feeders, choosing sound-producing fish disproportionately relative to their availability, as well as non-soniferous species [24,25]. For this study, we sought prey fish of 11 different species: Gulf toadfish (*Opsanus beta*), spot (*Leiostomus xanthurus*), spotted seatrout (*Cynoscion nebulosus*), pinfish (*Lagodon rhomboides*), sheepshead (*Archosargus probatocephalus*), striped mullet (*Mugil cephalus*), ladyfish (*Elops saurus*), pigfish (*Orthopristis chrysoptera*), Atlantic thread herring (*Opisthonema oglinum*), scaled sardine (*Harengula jaguana*), and hardhead catfish (*Ariopsis felis*). Additionally, the collected fish were between 12 cm and 64 cm in total length to ensure sufficient tissue volume for analyses. Fish samples for this project came from seasonal fish surveys in Sarasota Bay during September 2022, as part of long-term abundance monitoring [26]. Sampling occurred via state-approved licensing (Florida Fish and Wildlife Conservation Commission Special Activity License nos. 19-0809A-SR) and according to protocols approved by Mote Marine Laboratory’s Institutional Animal Care and Use Committee (IACUC). Whole fish were wrapped in solvent-rinsed foil and stored frozen at −20 °C until dissection. Prior to dissection, all fish were thawed and removed from the aluminum foil. Total length (cm), total mass (g), gastrointestinal tract mass (g), and muscle mass (g) were collected. For each fish, the entire gastrointestinal tract (GIT) and muscle tissue from one lateral side were removed using stainless steel instruments and stored separately in glass jars at −20 °C. Dissection trays, petri dishes, and instruments were rinsed three times with purified water (Milli-Q^®^) (Millipore, Molsheim, France) prior to dissection and tissue collection.

### 2.2. Dolphin Sampling

Bottlenose dolphin gastric sampling and analysis were previously described in [18]. Briefly, samples were collected during catch-and-release health assessments, in which dolphins were encircled using a net in shallow water, temporarily restrained, and brought on board a specialized, padded, and shaded sampling vessel for morphological and physical examination by veterinarians. During the examination, gastric samples were collected by passing a small veterinary feeding tube through the esophagus into the stomach [27–29]. Samples were stored in glass jars and frozen at −20 °C until particle analysis. Sarasota Bay bottlenose dolphin health assessments were conducted under permit from the National Oceanic and Atmospheric Administration’s (NOAA) National Marine Fisheries Service (NMFS) and IACUC-approved protocols.

### 2.3. Microplastic Screening

Microplastic screening of fish tissues followed methods used in [18,30]. Briefly, muscle and gastrointestinal (i.e., stomach and intestine) tissue from each fish were placed into a glass beaker, and organic (non-plastic) material in the samples was digested by adding a 10% potassium hydroxide (KOH) solution and incubated at 60 °C for 24–72 h [31]. Following digestion, samples were vacuum filtered onto GF/A 1.6 μm glass fiber filters in a fume hood and left to dry in covered glass petri dishes.

Particles of at least 35 μm were characterized visually using a dissection microscope (Leica EZ4, magnification 8–35×) according to physical attributes including shape (e.g., fiber, film, fragment, foam), surface texture (e.g., smooth, rough, rubber), and color (e.g., transparent, blue, black; [32]). Various parameters were used to identify potential plastic materials. For example, suspected plastic fibers were indicated by a smooth, uniform surface with a length that exceeded the width [33]. Suspected plastic fragments were characterized by smooth or angular edges that appeared to be broken from a larger piece of debris [33]. Fragments were further characterized as suspected tire wear particles (TWPs) if they were black, cylindrical, had a rubbery surface texture, and maintained their shape when manipulated with forceps [34]. Suspected foam particles were characterized by a round shape and honeycomb-like porosity [35]. Suspected fiber bundles were characterized by 20 or more fibers tangled together in a way that prevented them from being separated [36,37]. All particles at least 100 μm in size and with characteristics previously described were tested with a hot needle (250 °C) and suspected to be of plastic origin if the needle left a mark on or melted the particle surface [34,38,39]. Fourier transform infrared (FTIR) spectroscopy was available for polymer identification of particles ranging from 500 μm to 5 mm.

### 2.4. Quality Assurance/Quality Control

Rigorous precautions were taken while handling and processing samples. A 100% cotton lab coat and nitrile gloves were worn during fish dissections and laboratory analyses. All tools and glassware were carefully rinsed with deionized water prior to use. For QA/QC purposes, analysis of one “laboratory blank” (i.e., non-tissue sample) was performed simultaneously with each set of sample digestions to correct for potential procedural contamination, and three positive controls with commercially purchased polyethylene, polystyrene, and polyester microplastic particles were used to determine recovery efficiency. Mean recovery percentages for fish were 60% for film, 83% for foam, and 85% for fibers, while mean recovery percentages for dolphins were 90% for film, 87% for foam, and 90% for fibers [18]. Additionally, during fish dissections, an open petri dish was kept on the top of the bench to collect potential environmental contaminants and processed using the same methods as the fish tissues (“dissection blank”).

### 2.5. Statistical Methods

Descriptive statistics were used to calculate the percentage of fish with suspected plastics detected in either muscle or GIT samples, overall, by species, and by tissue type (i.e., muscle vs. GIT). The particle load for fish was calculated as the number of suspected plastic particles per gram of tissue, and these concentrations were compared between tissue types and species. Similarly, suspected plastic attributes (i.e., color and shape) were compared between tissue types and species. Finally, particle attributes were qualitatively compared with ingested microplastics in bottlenose dolphins reported in [18].

## 3. Results

**Figure 1.**
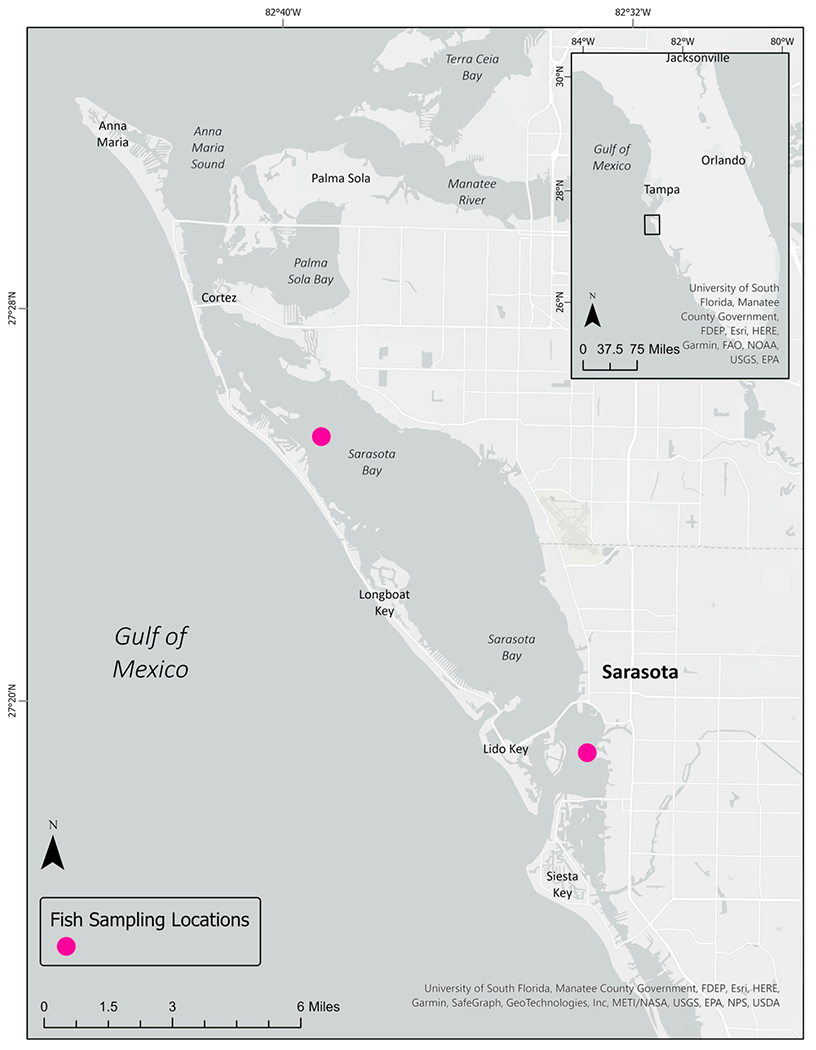
Locations of fish sampling in Sarasota Bay, FL, USA.

In total, 29 fish across four species (hardhead catfish (*n* = 2); pigfish (*n* = 12); pinfish (*n* = 10); and Gulf toadfish (*n* = 5)) were collected from two sampling stations in Sarasota Bay, FL, during September 2022 (Figure 1). All fish were screened for microplastics in either muscle (*n* = 4), GIT tissue (*n* = 3), or both (*n* = 22). Ninety-seven percent of the fish screened (*n* = 28) had evidence of at least one suspected plastic particle in either muscle or GI tissue. Species counts and mean tissue mass are reported in Table 1.

### 3.1. QA/QC Results

Suspected plastic particles were identified in both laboratory and dissection blanks (Table 2). Single fibers dominated the particles observed in the dissection blanks (*n* = 115), and major colors included transparent (*n* = 45), blue (*n* = 28), yellowed (*n* = 13), and pink/purple (*n* = 9) or orange (*n* = 9). “Yellowed” refers to fibers with a darker yellow appearance, likely discolored during the digestion process or particle degradation [40]. Transparent (*n* = 1) and yellowed (*n* = 1) films were also observed in dissection blanks, as well as yellowed (*n* = 1) non-TWP fragments. Single fibers (*n* = 60), one yellow film, and one red, non-TWP fragment were observed in laboratory blanks, with most fibers transparent (*n* = 19), orange (*n* = 12), or blue in color. Suspected plastic particles identified in muscle and GIT tissue samples were corrected for dissection and laboratory blank contamination by removing particles of the same shape and color from total particle counts.

### 3.2. Suspected Plastics in Prey Fish Muscle Tissue

Among blank-corrected muscle samples (*n* = 26), suspected plastic particles were observed in 77% of the fish evaluated (*n* = 20). Overall, the number of observed particles per fish ranged from 0 to 40; the highest count was observed among pigfish. The average number of particles per species was: hardhead catfish (3.0), pigfish (8.0), pinfish (3.8), and Gulf toadfish (5.6). Pigfish had the highest average suspected plastic particle load (2.9 particles/g tissue), while catfish had the lowest (0.2 particle/g tissue; Table 3). Of the 172 total particles observed in muscle tissue across all species, 90.1% were single fibers (Table 3). Other suspected particle types observed in muscle tissue included films (*n* = 1), non-TWP fragments (*n* = 1), and TWP fragments (*n* = 15; Table 3). Foam and fiber bundles were not observed in the muscle tissue of any fish examined. Of the 155 fibers observed across all species, most were either bright yellow in color (34.2%) or transparent that yellowed with time or processing (27.7%). The majority of total tire wear particles (*n* = 15) were observed in catfish (26.7%) or Gulf toadfish (60.0%; Table 3). All observed suspected particles in muscle tissue were less than 500 μm in diameter; thus, FTIR was not conducted to determine polymer composition.

### 3.3. Suspected Plastics in Prey Fish Gastrointestinal Tracts

Among blank-corrected GIT samples (*n* = 25), 88.0% of fish examined had at least one suspected plastic particle. Among GIT samples, suspected plastic particle counts ranged from 1 to 67, with pigfish having the highest count. By species, the average number of particles was hardhead catfish (9.0, *n* = 1), pigfish (16.1), pinfish (10.8), and Gulf toadfish (15.2). Gulf toadfish had the highest average suspected plastic particle load (3.2 particles/g tissue), while hardhead catfish and pinfish had the lowest (0.7 and 0.9 particle/g tissue, respectively; Table 4). Across all fish, 343 suspected particles were observed, and most were films (36.4%), followed by fiber bundles (29.2%), single fibers (28/3%), TWP fragments (2.9%), and non-TWP fragments (1.7%; Table 4). Similarly to muscle samples, foams were not observed in any fish GI tissue. Most films were transparent (82.4%), and other colors observed were blue (*n* = 1), red (*n* = 8), and yellowed (*n* = 13). TWP fragments were most common among pigfish (50% of observed) and not observed in the GI tract of the single catfish or most pinfish samples (Table 4). Most observed suspected particles in muscle tissue were less than 500 μm in diameter; thus, FTIR was not able to be conducted to determine polymer composition.

### 3.4. Comparison of Suspected Plastics in Fish and Bottlenose Dolphins

**Figure 2.**
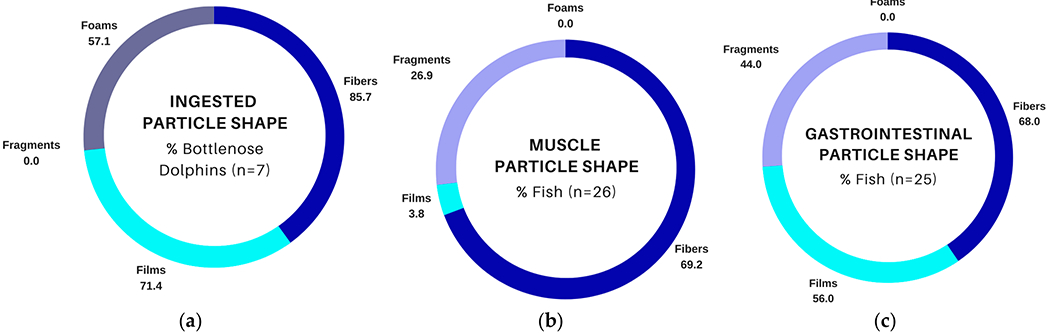
Comparison of suspected plastic particles (blank-corrected) in fish and bottlenose dolphins (*Tursiops truncatus*) sampled in Sarasota Bay, FL, USA: (**a**) percentage of dolphin gastric samples with at least one of the listed particle shapes, data from [18]; (**b**) percentage of fish muscle samples with at least one of the listed particle shapes; (**c**) percentage of fish gastrointestinal tract samples with at least one of the listed particle shapes.

Suspected plastic particles in muscle and GIT samples from fish collected from Sarasota Bay, FL, USA, were compared with ingested particles from bottlenose dolphins inhabiting the same area, as reported in [18]. In general, all sampled dolphins (*n* = 7) had at least one suspected plastic particle detected in gastric fluid, and particle counts exceeding 50 for an individual dolphin were common [18]. Dolphin samples contained primarily fibers, films, and foams (Figure 2a); however, foams were not observed in muscle or GIT tissues from fish (Figure 2b,c). Fibers and fragments were the dominant particle shapes observed in fish muscle (Figure 2b), while fish GIT samples contained a mixture of fibers, films, and fragments (non-TWP and TWP; Figure 2c). TWP fragments were not observed in bottlenose dolphin gastric samples, but 23.1% and 32.0% of muscle and GIT samples, respectively, contained at least one TWP fragment.

**Figure 3.**
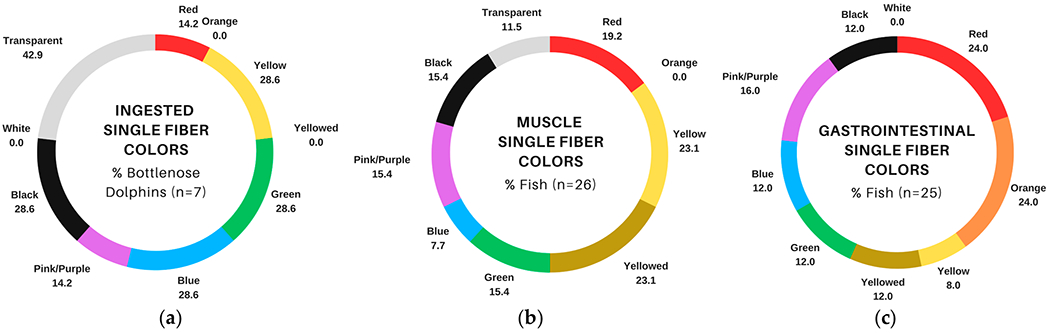
Comparison of single fiber colors (blank-corrected) in fish and bottlenose dolphins (*Tursiops truncatus*) sampled in Sarasota Bay, FL, USA: (**a**) percentage of dolphin gastric samples with at least one of the listed fiber colors, data from [18]; (**b**) percentage of fish muscle samples with at least one of the listed fiber colors; (**c**) percentage of fish gastrointestinal tract samples with at least one of the listed fiber colors.

Fibers were common in bottlenose dolphin gastric samples and both types of fish tissues (Figure 2a–c); however, there were differences in the distribution of colors. Transparent fibers were most common in dolphin samples (42.9% of samples; Figure 3a), while brighter colors such as yellow/yellowed, red, and orange were more abundant among fish samples (Figure 3b,c). Single fiber counts were more numerous in fish (Tables 3 and 4) compared to dolphins [18], and fiber bundles that were present in fish GIT samples (Table 4) were not reported in bottlenose dolphins [18].

**Figure 4.**
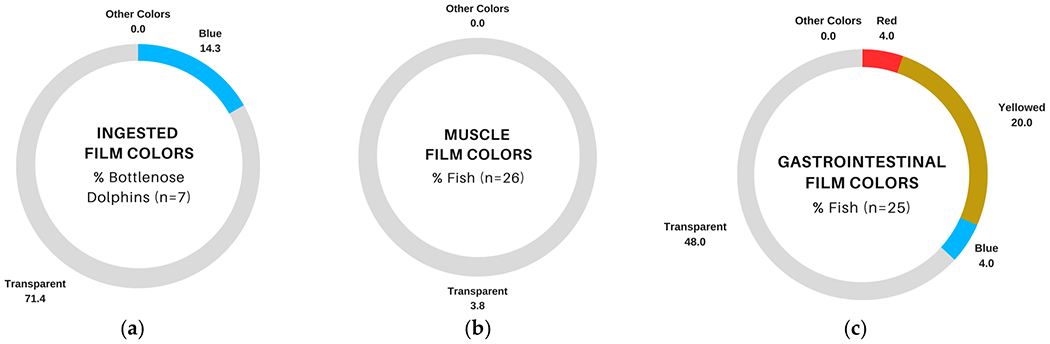
Comparison of film colors (blank-corrected) in fish and bottlenose dolphins (*Tursiops truncatus*) sampled in Sarasota Bay, FL, USA: (**a**) percentage of dolphin gastric samples with at least one of the listed film colors, data from [18]; (**b**) percentage of fish muscle samples with at least one of the listed film colors; (**c**) percentage of fish gastrointestinal tract samples with at least one of the listed film colors.

Films were also observed in dolphin gastric samples (71.4%; Figure 2a) and both fish tissue types, although they were much less abundant in muscle (3.8%; Figure 2b) than in GIT (56.0%; Figure 2c). Most films in bottlenose dolphin gastric samples and fish samples were transparent (Figure 4a–c), although 20.0% of fish GIT samples also contained yellowed films, which were not observed in bottlenose dolphins (Figure 4a,c).

## 4. Discussion

This study provides additional evidence of exposure to suspected plastic particles among marine fauna inhabiting an urban estuary in Sarasota Bay, FL, USA. Sarasota Bay is located on the central, west coast of Florida and is a semi-closed lagoon system with minimal tidal exchange [41]. As an urban watershed, this region houses multiple residential, industrial, and commercial centers [42]. Findings from this study are not surprising as plastic ingestion in marine fauna has been well documented across multiple taxa, including bivalves [43–45], fish [46–49], and marine mammals (e.g., pinnipeds, [50–52]; mysticetes, [8,53,54]; odontocetes, [55–57]). Additionally, waters surrounding urban centers are generally more polluted than rural areas [58–60], thereby increasing exposure to plastic debris for aquatic animals that live in close proximity to urbanized areas [48,61–63].

Among the four fish species investigated in this study, suspected plastic particles were observed in both muscle and GIT tissue/contents, with particles more abundant in GIT. We expected to find suspected plastic particles in the gastrointestinal tract based on previous work [62,64–67], similar to researchers who have studied intentional/selective [68,69] and unintentional [70] ingestion. Observations of particles in muscle tissue were also not surprising based on previous studies in wild-caught fish from the Persian Gulf [71,72], India [73], and the Mediterranean Sea [74], as well as experimental evidence by [75], who demonstrated in juvenile seabass (*Dicentrarchus labrax*) that particles can be translocated to muscle tissue. These authors suggest that translocation to muscle tissue likely occurs via lymphatic or vascular systems, is restricted to small particle sizes, and likely explains the differences in total particle counts observed between muscle and GIT tissues [75].

Our comparison of ingested particles in dolphins with suspected particles observed in four prey species (hardhead catfish, pinfish, pigfish, and Gulf toadfish) revealed both similarities and differences in particle attributes. Single fibers were common in bottlenose dolphin samples and fish tissues, but the distribution of colors differed, with transparent fibers dominating dolphin samples and bright colors dominating single fibers in fish samples. Among marine fauna, ingestion of microplastics can be both indirect (i.e., ingesting microplastic-contaminated prey; [50,76,77]) and direct (i.e., ingesting plastics from the water column; [13,46,68]). Planktivorous fish species that hunt via sight can mistake microplastics for prey that are similar in size and color [13]. For example, in [46], red particles were frequently observed in omnivorous fish that commonly consume red algae, and [68] demonstrated blue particle selectivity among amberstripe scads (*Decapterus muroadsi*), which commonly eat blue copepods. It also seems possible that the color of ingested fibers could change or fade over time due to chemical degradation [40] occurring in the digestive tract. In other words, yellowed or transparent fibers could have originated as a different color. With respect to the potential for trophic transfer of fibers between fish and dolphins, slow egestion rates of fibers in fish [78] could render them available for incidental consumption by foraging dolphins.

Transparent films were also commonly observed in dolphin and fish samples. This particle shape has been widely reported in previous studies of fish exposed to microplastics [79–83], and given evidence of trophic transfer of other microplastic shapes [50], it seems plausible that contaminated fish could be the source of transparent films for bottlenose dolphins. TWP fragments were not observed in any dolphin sample, but they were observed in approximately 23.1% and 32.0% of fish muscle and GIT tissues, respectively. TWP fragments have been observed in multiple matrices (e.g., air, water, sediment, and biota (reviewed by [84])) and geographic locations (e.g., Charleston Harbor (USA) [34], San Francisco Bay (USA) [85], and the Seine (France), Chesapeake (USA), and Yoda (Japan) watersheds [86]). So, given the preponderance of these fragments in fish tissues, it is somewhat surprising that they were not observed in the gastric fluids of bottlenose dolphins from Sarasota Bay [18]. One possible explanation is that TWP fragments have a higher propensity to become entrapped in the mucus lining the gastrointestinal tract than other microplastic particles. Supporting this notion are observations regarding the fate of nanoparticles in isolated intestines, which found the bulk of particles became trapped in the mucus lining, and no particles were found in the gastric fluids of the lumen or adhered to the epithelium [87]. Certainly, further research is warranted concerning the fate of microplastic particles, including TWPs, in the gastrointestinal tracts of mammals. White foams were the most abundant particle observed in bottlenose dolphin gastric samples, sometimes in quantities exceeding 50 per sample [18]. White foams were not present in either fish tissue. Possible explanations for this difference could be the relatively small number of fish evaluated for each species or the small number of species screened; additional sampling of fish within and across more species could help to discern whether the ingested foams in dolphins could be related to contaminated prey. Alternatively, it could be possible that dolphins accidentally ingest foams if they engage in object play behavior [88,89] or physically manipulate larger, floating foam objects (e.g., food and drink containers, buoys).

One of the limitations in microplastic research is the recurring issue of sample contamination by ambient microplastics during sample processing and analysis [90]. Rigorous precautions were taken while handling and processing samples. Additionally, conservative approaches to blank-correct particle counts in fish tissues were performed such that particle shapes and colors observed in blanks were removed from the tissue counts of associated fish. Despite these stringent measures to minimize contamination, we were unable to confirm the polymer composition of particles from muscles and GITs. Fourier transform infrared spectroscopy (FTIR) is an instrumental technique used to identify functional groups using light refraction [91], but FTIR’s utility is limited to particles ranging from 500 μm to 5 mm. Most microscopy-identified particles in this study were <500 μm; thus, most of the reported particle counts are based on hot needle testing [39]. Sample size is another limitation impacting our ability to trace ingested microplastics in bottlenose dolphins to particles observed in prey fish. Hart et al. (2022) evaluated gastric samples for seven individuals and observed abundant white foams, which were not present in any fish sample screened for this study. While these findings might suggest that dolphins are not exposed to white foam particles via trophic transfer, it is also possible that we did not sample enough fish, overall or within a species, to detect white foams, as they have been observed in other estuarine fish [67]. Small sample sizes also limited our ability to quantitatively compare particle counts and characteristics between species and tissue types. Additional bottlenose dolphin and fish samples will be collected to further explore evidence of trophic transfer and the species-level risk of plastic exposure for bottlenose dolphins. Recent efforts have been made to enhance our understanding of marine mammal diets using quantitative fatty acid signature analysis (QFASA; [92]). These techniques compare fatty acid profiles in tissue from a predator with a database of fatty acid profiles in common prey species so that researchers can identify the primary components of an individual’s diet [92]. Blubber samples for fatty acid analyses were also collected from Sarasota Bay bottlenose dolphins, so future studies could use these techniques and species-level microplastic counts (for fish) to understand individualized diets and conduct individualized risk assessments.

Marine plastic debris is now recognized as a pollutant of international concern due to its impacts on wildlife and seafood safety. Eriksen et al. (2023) estimated that the oceans contain more than 171 trillion plastic particles, and most (92.4%; [3]) are microplastics. Given that an estimated 41% of the world’s population lives within 100 km of the coast [93], this widespread marine pollutant may have substantial public health consequences. Observations of microplastics in the tissues of wild-caught fish and gastric samples of estuarine bottlenose dolphins may warn of local environmental pollution and seafood safety risks for coastal communities.

## Figures and Tables

**Table 1.** Mean muscle and GIT mass (g) by fish species.

Species	Sample Size	Muscle Mass (g) Mean (s.d.)	GIT Mass (g) Mean (s.d.)
Hardhead catfish (*Ariopsis felis*)	2	16.3–23.2 ^1^	12.8 ^1^
Pigfish (*Orthopristis chrysoptera*)	12	2.8 (0.8)	6.4 (2.1)
Pinfish (*Lagodon rhomboides*)	10	3.8 (1.3)	12.0 (7.0)
Gulf toadfish (*Opsanus beta*)	5	4.1 (3.0)	4.8 (1.9)

1 Actual values reported due to small sample size.

**Table 2.** Suspected plastic particles observed in laboratory and dissection blanks.

Blank Type	n	Single Fibers	Fiber Bundles	Films	Fragments (TWP)	Fragments (Non-TWP)	Foams
Dissection	6	115	0	2	0	1	0
Laboratory	11	60	0	1	0	1	0

**Table 3.** Suspected plastic particle counts by shape in blank-corrected fish muscle samples (*n* = 26). Particle load reported as number of particles (#) per gram of tissue.

Species	Sample Size	Total Particles	Particle Load (#/g Tissue)	Single Fibers	Fiber Bundles	Films	Non-TWP Fragments	TWP Fragments
Hardhead catfish (*Ariopsis felis*)	2	6	0.2	2	0	0	0	4
Pigfish (*Orthopristis chrysoptera*)	11	88	2.9	86	0	0	1	1
Pinfish (*Lagodon rhomboides*)	8	50	1.6	49	0	0	0	1
Gulf toadfish (*Opsanus beta*)	5	28	1.4	18	0	1	0	9
Total	26	172	-	155	0	1	1	15

**Table 4.** Suspected plastic particle counts by shape in fish gastrointestinal samples (*n* = 25). Particle load reported as number of particles (#) per gram of tissue.

Species	Sample Size	Total Particles	Particle Load (#/g Tissue)	Single Fibers	Fiber Bundles	Films	Non-TWP Fragments	TWP Fragments
Hardhead catfish (*Ariopsis felis*)	1	9	0.7	1	0	3	0	0
Pigfish (*Orthopristis chrysoptera*)	10	161	2.5	32	44	77	3	5
Pinfish (*Lagodon rhomboides*)	9	97	0.9	36	34	23	2	2
Gulf toadfish (*Opsanus beta*)	5	76	3.2	22	28	22	1	3
Total	25	343		97	100	125	6	10

## Data Availability

The fish data (used for this manuscript) can be accessed using this DOI link: https://doi.org/10.5061/dryad.fn2z34v1d. Bottlenose dolphin data can be accessed using the repository cited in the original paper (Hart et al., 2022), doi: 10.3389/fmars.2022.947124.
